# Experiences of fear of falling in persons with Parkinson’s disease – a qualitative study

**DOI:** 10.1186/s12877-018-0735-1

**Published:** 2018-02-06

**Authors:** Stina B. Jonasson, Maria H. Nilsson, Jan Lexell, Gunilla Carlsson

**Affiliations:** 1 Department of Health Sciences, Lund University, Box 157, 221 00 Lund, Sweden; 2 Department of Neurology and Rehabilitation, Skåne University Hospital, Lund, Sweden; 3 Memory Clinic, Skåne University Hospital, Malmö, Sweden; 4 Department of Health Sciences, Luleå University of Technology, Luleå, Sweden

**Keywords:** Parkinson disease, Self efficacy, Accidental falls, Qualitative research, Interview

## Abstract

**Background:**

Fear of falling is common among persons with Parkinson’s disease and is negatively associated with quality of life. However a lack of in-depth understanding of fear of falling as a phenomenon persists. This qualitative study aimed to explore the experiences of fear of falling in persons with Parkinson’s disease.

**Methods:**

Individual interviews were performed with twelve persons with Parkinson’s disease (median age 70 years, median Parkinson duration 9 years, 50% women). The interviews were semi-structured and followed a study-specific interview guide. The transcribed interviews were analyzed using qualitative content analysis.

**Results:**

Fear of falling was experienced as a disturbing factor in everyday life. It generated a feeling of vulnerability and made daily activities and everyday environments seem potentially hazardous. Persons also missed performing previous activities. The fear of falling was a varying experience, fueled by an awareness of falls and near falls, Parkinson-related symptoms and disabilities, and by others in their environment. The persons adopted different strategies to handle their fear of falling. Activities were adapted, avoided, performed with help, or carried out despite their fear of falling.

**Conclusions:**

The experiences of fear of falling were complex, multifaceted and varied over time and in relation to different activities and environments. This indicates that interventions targeting fear of falling need to be individually tailored for persons with Parkinson’s disease and should focus on several aspects, such as Parkinson-related symptoms and disabilities, activities and environmental factors. This study provides new information that increases the understanding of fear of falling, which has implications for researchers as well as clinicians working with persons with Parkinson’s disease and fear of falling.

**Electronic supplementary material:**

The online version of this article (10.1186/s12877-018-0735-1) contains supplementary material, which is available to authorized users.

## Background

Fear of falling (FOF) is a common and non-negligible problem in persons with Parkinson’s disease (PD) [1–4]. In a previous study, 11% of the participants with PD described FOF as their most stressful physical symptom [5]. FOF is a predictor of future falls and near falls already in mild PD [6], and is negatively associated with participation and health-related as well as overall quality of life [7–9]. Studies that used multivariable regression analyses to identify explanatory factors of FOF have shown that PD-related disabilities such as walking difficulties, need of help in activities of daily living, motor symptoms, orthostatism, poor functional balance and fatigue are significantly associated with FOF in people with PD [2, 4, 8, 10–13]. Female gender and a history of falls have been shown to be significant and independently explanatory factors of FOF in the overall elderly population [14] but not in people with PD [2, 4, 10, 11, 13]. Beside these above pre-defined variables, there might be other variables of importance in relation to FOF as perceived by persons with PD.

So far, most studies that addressed FOF have used a quantitative design. Qualitative PD studies that focused on concepts other than FOF described how it negatively impacts on activities and perceived participation [9, 15] and is seen as a great loss of freedom [16]. There are qualitative studies of FOF in elderly persons without PD [17–22]. However, since many explanatory factors of FOF in persons with PD are PD-related [2, 4, 8, 10–13], it is plausible that the experience of FOF in persons with PD differ to those without PD. To the best of our knowledge, no study has explored how persons with PD experience FOF. Such knowledge would be of value for the clinical care of persons with PD. That is, an increased knowledge of the experiences of FOF could facilitate the support of persons with PD to handle their FOF as well as the treatment of persons with PD and FOF. Thus, the aim of this study was to explore the experiences of FOF in persons with PD who have reported FOF.

## Methods

This qualitative study is based on semi-structured individual interviews with twelve persons with PD. Individual interviews are considered suitable for examining peoples’ views and experiences on sensitive topics [23], such as FOF.

### Recruitment of participants

Interviewees were recruited among participants that previously have participated in a postal survey study [3]. Data for that study were collected during the spring of 2013 and included 102 persons with a clinically confirmed PD diagnosis (ICD-10: G 20.9) who were recruited from two outpatient hospital clinics in southern Sweden (for more details, see [3]). Potential participants for the present study were selected among the 56 persons who in 2013 stated that they were afraid of falling (i.e., answered “Yes” on a dichotomous question on FOF). A strategic selection was performed based on data from the postal survey study, striving for heterogeneity regarding age, gender, PD severity, degree of FOF (assessed by the Falls Efficacy Scale-International [24]), living circumstances, previous falls and the use of mobility devices.

Initially, 25 potential participants were contacted. As the quantitative data collection (described below) revealed too few persons with a high level of FOF, an additional six potential participants (who reported high levels of FOF in the postal survey study) were contacted. All potential participants received information about the study and an invitation letter, and were contacted by phone by one of the authors (SBJ). Ten persons declined to participate, three could not participate due to health-related issues and one was excluded since he did not experience any FOF. A total of 17 persons agreed to participate. During the phone call, these 17 persons were asked questions which constituted the basis for final selection of participants. The questions targeted PD duration, self-rated PD severity (mild/moderate/severe), intensity of FOF (are you afraid of falling: not at all/a little/somewhat/very much [25]), frequency of FOF (are you afraid of falling: never/almost never/sometimes/often/very often [26]), living circumstances (alone/not alone), occurrence of falls during the past six months and the use of mobility devices.

In order to obtain a heterogenic study sample with various experiences of FOF, the final selection of participants was based on the responses to the questions posed during the phone call (described above). The selection of participants was performed in several steps to ensure the inclusion of information rich cases in relation to the experiences of FOF. The inclusion process continued until no new data of interest for the study aim emerged during the qualitative interviews. This resulted in the inclusion of twelve persons. At the time when the researchers had decided to terminate the data collection, the five non-participants were contacted and informed about the decision not to include them in the study.

### Procedure

The twelve participants were contacted in order to schedule dates and times for interviews, according to their preferences. All interviews were conducted between July and October 2014 in the participants’ homes by the same interviewer (SBJ, first author) with no other person present. Before the interviews, the interviewer again informed the participants about the aim of the study and the participants gave their written informed consent. The interviews were semi-structured and followed a study-specific interview guide, which had been tested and refined during three test interviews with persons with PD and FOF (not included in the present study). The interview guide contained open-ended questions that enabled the participants to talk freely about, for example, the importance and meaning of FOF, activities and situations when FOF was experienced, variations in FOF due to external and internal factors, and the perceived consequences of FOF (see Additional file 1). Follow-up questions and probes were used to deepen the participants’ answers. At several occasions during the interviews, the participants shifted focus and talked in-depth about their experiences of falls, and the interviewer sometimes had to lead the interviews back to the subject, i.e., FOF.

The interviews were audio recorded and lasted from 25 to 78 (median 40) minutes. Field notes were taken during the interviews. At the end of each interview, the researcher summarized the main points based on the field notes, and gave each participant the opportunity to add comments and clarifications.

Directly after the interviews, the participants again responded to questions that addressed PD severity, living circumstances, intensity of FOF, falls during the past six months, and the use of mobility devices in order to get updated information on these variables. Furthermore, the participants responded to questions regarding the occurrence of dyskinesia (yes/no), occurrence of fluctuations with increasing PD symptoms (yes/no), and difficulties in activities of daily living (Parkinson’s disease Activities of Daily Living Scale [27]). Their cognitive function was assessed using the Montreal Cognitive Assessment [28].

The interviews were transcribed verbatim by the interviewer. After four interviews, the first and the last author (who has extensive experience of qualitative studies) read the transcripts and discussed the interviews in order to ensure that no important areas were missed. Another six interviews were then conducted, and the transcripts were read and discussed by all four authors. After two additional interviews, the first and last author discussed all twelve interviews and found that no new data of interest for the study aim had emerged, and subsequently decided to terminate the data collection.

### Participants

The characteristics of the twelve participants (6 women, 6 men) are presented in Table 1. Their median age was 70 (first-third quartile 66–75, min-max 58–90) years. Median PD duration was 9 (first-third quartile 5–12, min-max 3–25) years. Five participants were a little afraid of falling, three were somewhat afraid, and four were very much afraid of falling. Nine participants had experienced at least one fall during the past six months. Of these, five participants were single-fallers and four were recurrent fallers during the past six months: one participant reported two falls, one participant reported ten falls and two participants reported two falls every week (i.e., 52 falls). Eleven participants were community living and one participant lived in an assisted living facility. Four participants experienced dyskinesia and six experienced fluctuations with increasing PD symptoms. Although no participant rated their PD as severe at the time of the qualitative interview, three participants rated their PD as severe during the phone call at the initial contact.

**Table 1:** Participants’ characteristics at the time of the interviews, *n* = 12

Participant number	Gender	PD severity^a^	Cognitive function (MoCA)^b^	Need of help from others in daily activities^c^	Living alone	Fear of falling, intensity^d^	Falls past six months	Mobility device indoors	Dyskinesia	Motor fluctu-ations
1	Male	Moderate	23	Yes	No	Very much	Yes	Yes	No	No
2	Male	Moderate	25	No	No	A little	Yes	No	No	No
3	Female	Moderate	22	Yes	Yes	Somewhat	Yes	Yes	No	No
4	Female	Mild	28	No	No	A little	No	No	No	No
5	Male	Moderate	22	Yes	No	Somewhat	Yes	Yes	No	Yes
6	Female	Moderate	22	No	No	Somewhat	Yes	No	No	Yes
7	Male	Moderate	18	No	Yes	A little	Yes	Yes	Yes	Yes
8	Male	Moderate	24	No	Yes	Very much	No	Yes	Yes	Yes
9	Female	Mild	22	No	Yes	A little	Yes	No	Yes	No
10	Male	Mild	29	No	No	A little	No	No	No	No
11	Female	Moderate	23	Yes	No	Very much	Yes	Yes	Yes	Yes
12	Female	Moderate	24	Yes	No	Very much	Yes	Yes	No	Yes

*PD* Parkinson’s disease, *MoCA* Montreal Cognitive Assessment

^a^Self-rated. Possible response options: Mild, Moderate, Severe

^b^Possible scoring range: 0–30 (higher = better)

^c^Assessed by Parkinson’s disease Activities of Daily Living Scale. Dichotomized: “No” and “Mild difficulties with day-to-day activities” are recorded as not needing help from others. “Moderate”, “High levels of” and “Extreme difficulties with day-to-day activities” are recorded as needing help from others

^d^Possible response options: Not at all, A little, Somewhat, Very much

### Data analysis

The interviews were analyzed using manifest and latent content analysis conducted in several steps, as described by Graneheim and Lundman [29]. This is a well-structured methodological approach, suitable for exploring variations of a phenomenon. The twelve transcribed interviews constituted the units of analysis that were coded, categorized and interpreted. The first overall analysis of the interviews was performed gradually as the interviews were completed. The twelve interviews subsequently were read several times to obtain a sense of the whole. The text was divided into meaning units, which were then condensed and assigned various codes, describing the content. The codes were grouped into categories, which constituted descriptions of the manifest content of the interviews. After the categorization process, the analysis continued through interpretation of the categories into themes, representing the latent, underlying meaning of the interviews.

The researchers alternated between looking at the codes and studying the interviews in order to maintain a sense of the context. The first author, who has clinical experience of conducting anamnestic interviews, but limited experience of qualitative research interviews, conducted and transcribed the interviews and performed the initial analysis. Subsequently, the first author discussed the interviews, codes, categories and themes on several occasions with the three other authors, who are senior researchers with extensive research experience of qualitative interviews. The process of categorization and interpretation resulted in repeated adjustments and restructuring of categories and themes. These discussions mainly involved the first and last author but all authors were involved throughout the analytical phase, in order to ensure that categories and themes fitted the data and that the analysis addressed the intended focus of the study [29].

### Ethics

The study was conducted in accordance with the Helsinki Declaration and was approved by the Regional Ethical Review Board in Lund, Sweden (Dnr 2014/412). All participants gave their written informed consent. Before this study, the interviewer had mailed a postal survey to the participants in relation to a previous study [3] but had never met any of them and had no other professional relationship with them.

## Results

**Fig. 1:**
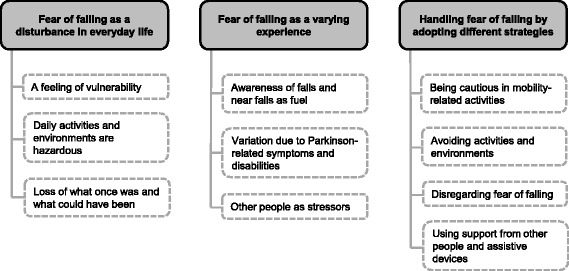
Overview of themes (grey boxes) and underlying categories (white boxes) of the experiences of fear of falling in persons with Parkinson’s disease

FOF affected everyday life in several ways and the participants’ experiences were diverse and expressed in many ways. Three themes that covered the experiences of FOF in persons with PD emerged from the data, namely: (i) FOF as a disturbance in everyday life; (ii) FOF as a varying experience; and (iii) Handling FOF by adopting different strategies. These themes are outlined below, and the structure of the themes and categories is described in Fig. 1.

### Fear of falling as a disturbance in everyday life

FOF was described as making everyday life daunting and less enjoyable. Many participants described that FOF was always at the back of their minds and gave them a feeling of insecurity during everyday activities. Several activities, situations and environments were described as potentially hazardous. Many participants described a longing to lead a normal life; a life without FOF was considered to be more active and would offer greater freedom. For some, FOF took the joy out of previously appreciated activities.

This theme contains three underlying categories, namely: A feeling of vulnerability; Daily activities and environments are hazardous; and Loss of what once was and what could have been. These categories are described below.

#### A feeling of vulnerability

Many participants described their FOF as always present at the back of their minds, although they did not actively think about it at all times. It was common to feel insecure in crowds and among people in a hurry, and many were afraid of getting bumped into.

I don’t like to visit supermarkets because there are so many people, and they are in a hurry and… Yes, you feel like you are in a world where you don’t… well, that you are too old for. (…) In supermarkets there are lots of people and everyone is rushing towards the check-out. Yes, then you are afraid of getting pushed, that is. (#9)

One woman was afraid of falling in her relatives’ fancy house, causing a mess. The participants expressed that their FOF made them feel nervous, insecure and vulnerable. They were afraid of not being able to get up after a fall.

Sometimes, I think about what would have happened if I had fallen here, or what if I had fallen… Nobody knows where I am. (…) I thought, if I had fallen… and had been lying there, then maybe I would have been lying there for a long time. (#4)

Many described that FOF increased while being alone and that they preferred to be accompanied by someone when leaving their house. One man expressed that, due to poor balance, he almost did not dare to do his home exercises due to the risk of falling. This fear was reduced while being at the physiotherapy clinic where there was always someone around.

#### Daily activities and environments are hazardous

The participants described that their FOF made a variety of activities and environments seem hazardous and daunting. Some mentioned FOF in regular activities, such as turning while standing, getting up from lying or sitting, reaching, leaning forward, and getting in and out of a car. The design of the physical environment was expressed to induce specific challenges, e.g., tactile paving (designed for visually impaired people) and narrow walkways were perceived as hazardous by some participants. Many participants mentioned that hard surfaces provoked their fear since a fall on these surfaces could result in a greater injury. Slippery and uneven surfaces were frequently mentioned, sometimes using words as “being terrified” in the shower because of the slippery floor. One woman said that her FOF increased when she was walking on shiny floors that looked slippery, even if they were not. Many had a greater FOF outdoors but the opposite was also expressed, e.g., since indoor environments implied more starts and stops which could induce freezing episodes specific for people with PD. Some described the home as a comfort zone with less FOF, although many had experienced most falls at home. Stairs (especially walking downstairs) were brought up as a source for FOF by a majority of the participants and some feared escalators. One man said that he had an “unpleasant feeling” when taking the first step down a flight of stairs and one woman said that she had feelings of panic if she had to use an escalator. However, some stated that they did not have any FOF when climbing stairs.

#### Loss of what once was and what could have been

Many participants expressed how their FOF restricted everyday life and sometimes took the joy out of previously appreciated activities. The participants believed that they would have more freedom, and be more active and more out and about in public if they did not experience FOF. Some stated that their FOF interfered with their social life and caused isolation. One woman expressed that she hesitated leaving her house due to her FOF and that she thereby missed out on a lot of things.

Yes, I think it [life without FOF] would have been different. I think it would. (…) Yes, it would have been much easier to live then. If you didn’t have to think about it. (#12)

Some participants missed activities that they could no longer do, e.g., bicycling. For some, it was evident that even though they could still take part in many activities, their FOF restricted them from fully participating. For example, one woman mentioned how her friends walked ahead of her and went down an escalator, while she had to go searching for an elevator since she did not dare to use the escalator.

### Fear of falling as a varying experience

The participants’ experiences of FOF were not constant. They were aware of the increased risk of falling when having PD, which impacted on their FOF. Apart from their own experiences, other’s experiences of falls augmented their fear. Moreover, FOF was impacted upon by PD-related symptoms and disabilities, and the attitudes and treatment from other people. FOF increased when people wanted to help but offered the help in a way that did not suit the person with PD.

This theme contains three underlying categories, namely: Awareness of falls and near falls as fuel; Variation due to Parkinson-related symptoms and disabilities; and Other people as stressors. These categories are described below.

#### Awareness of falls and near falls as fuel

Some participants described that their FOF had developed as they realized that falls are often part of the PD profile. Their fear increased since they knew that falls can occur suddenly and at all times. Most participants had experienced falls and some wondered when they would fall next time. They were afraid of the consequences of falls and many were afraid of potential injuries.

It’s getting injured [that makes me afraid of falling]. You can break your neck and all sorts of things if you have a bad fall. Larger injuries that is. Smaller bruises and stuff like that are alright but… But, if you get permanently injured, that’s not fun. (#1)

Two participants had seen others fall or heard people talking about their falls. Although these participants had experienced falls themselves, they expressed that their FOF had increased by seeing or hearing about other persons’ falls. FOF was also expressed in relation to near falls, i.e., situations where they almost fell but managed to regain balance. These situations were described as unpleasant and even shocking, and it made them feel shaken and insecure.

#### Variation due to Parkinson-related symptoms and disabilities

The participants expressed that their FOF was aggravated as their PD progressed, due to increased walking difficulties, hyperkinesia, rigidity, wearing off episodes (i.e., episodes with worsened PD-related symptoms), freezing of gait and impaired balance. The general opinion was that how you feel at the moment affects your FOF. It was perceived as uncomfortable not being in full control of yourself. Many participants described that their FOF increased at times when they were feeling low, tired or stressed, while they had less or no FOF at times when they were in good spirits.

I would guess that when you are in good spirits, then the concerns about falling are reduced, or gone. And if you are feeling down then, then all the… dark thoughts will appear and then you notice both this and that, that is hazardous. (#7)

One man expressed how his worries about falling were more or less gone since he had better control over his body after deep brain stimulation surgery.

#### Other people as stressors

The participants expressed that their FOF sometimes increased due to the attitudes and treatment from other people. They described that people wanted to help, but the help could be “too much”. There was a need to manage by oneself and their FOF increased if someone tried to help but did it in the “wrong way”. One woman said that some people in the home care service grabbed her wheeled walker in a rough way when she walked, which increased her FOF. One man mentioned how people on the street rushed to help him, grabbed him in order to help, but they did not know how and thereby worsened the situation and increased his FOF. Some expressed that people stressed them in various situations. The participants thought that people had certain expectations and they felt obliged to do things faster than they had preferred, which negatively affected their FOF.

### Handling fear of falling by adopting different strategies

All participants used strategies in one way or another to handle their FOF. Activities were performed with more caution than before and the participants thought actively about how they walked and moved around. Some activities and environments were avoided altogether since their FOF was too intense in those situations. Other activities were performed despite their FOF, e.g., because the participants thought that they “had to do” certain activities, or their FOF was distracted by doing something they valued. Other ways to handle FOF was by obtaining help from other people or by using assistive devices.

This theme contains four underlying categories, namely: Being cautious in mobility-related activities; Avoiding activities and environments; Disregarding FOF; and Using support from other people and assistive devices. These categories are described below.

#### Being cautious in mobility-related activities

FOF made the participants wary and attentive at all times, looking for and registering risks in the environment. One man said that he used to look for safe directions to fall. Many described how activities and motions were performed at a slower pace and with more caution due to their FOF, especially if they were alone or in an environment that felt unsafe.

I guess I’m a little more cautious [due to my worry for falling]. Um, yes. It’s not the same… action and playing around. …Yes, I’m more cautious. (#10)

Many participants mentioned that their FOF had made them adapt their pattern of movement. Some actively thought about walking and moving “properly”, including, e.g., increasing foot clearance during gait. Raised attentiveness while climbing stairs, especially if carrying something at the same time, was commonly expressed. Some said that they used tricks to avoid or overcome freezing episodes and that this reduced their FOF.

It [my FOF] means that I always have to think twice. I say to myself, stand properly! (#3)

#### Avoiding activities and environments

Due to FOF, activities and environments that were perceived as unsafe were avoided to a varied extent. Some participants mentioned that they rarely visited stores and shops due to an increased FOF in these environments. Many avoided carrying things while climbing stairs and some avoided stairs altogether due to their FOF. Inclement weather such as snow and ice, as well as slippery walkways were often avoided since these conditions considerably increased FOF.

You try to live as before but, you… notice, quite a lot, there are things that you can’t do. To fix something on the roof of the house for example. Afraid of, if you fall there, then you injure yourself really badly, right. You avoid those situations, where a fall could cause real injuries. (#2)

#### Disregarding fear of falling

The participants endeavored to lead a normal daily life as much as possible and thereby handle their FOF. The desire to do something was sometimes prioritized above the FOF. This meant that some activities were performed despite an ongoing FOF, simply because the participants wanted to. Some said that they went out for a walk although they were afraid of falling, “hoping for the best”. Some stated that their FOF could be distracted by having other things to concentrate on, or by doing something that was perceived as enjoyable; at such times they did not think about their fear.

If it’s a new store that I visit, then I don’t think that much about the worry. It’s these kinds of aspects as well. (…) In stores, there are so much to look at. Then the worry disappears! Because then you have something else to think of. (#8)

Several participants said that they “had to do” certain things, despite experiencing FOF in those situations (e.g., leaving the home or climbing stairs).

Well it’s rare [that I climb stairs]. It’s difficult. When I do I’m really worried. (…) Sometimes you have to go up to the attic. And that, then I’m really scared. (#8)

Some performed activities as a training practice, despite their FOF. One man mentioned positive thinking and self-efficacy, that you can do more than you think. Another one said that a higher self-confidence would reduce his FOF. Some participants could neglect their FOF and stated that they were not affected by it.

#### Using support from other people and assistive devices

Most participants expressed that company and support from people reduced FOF if it was provided according to their needs and wishes. Some said that their family encouraged and provided support so that they carried out activities despite their FOF.

He [my husband] can, if he stands right in front of me [in the escalator] and sort of holds me… It’s not necessary for him to hold me tight or anything, it’s only so that I have something that I can grab hold on. If it wasn’t my husband I guess it would do with someone else as well, who knows how to do it. (#4)

A variety of assistive devices were described that reduced FOF, but the participants also identified shortcomings that increased their fear. Several participants expressed that their security alarm (connected to home care services) provided a sense of security but also pinpointed that the alarm did not work outdoors. Walking devices were used by many participants and perceived to reduce their FOF, but they were not considered to be entirely safe. Several participants expressed that wheeled walkers can tip over or roll away when least expected. Other assistive devices that were mentioned to reduce FOF were handrails in stairs, an access ramp (for avoidance of stairs) and a dishwasher (reduces time for standing and doing the dishes).

## Discussion

This is, to the best of our knowledge, the first study that has focused on the experiences of FOF in persons with PD. We found that FOF was experienced as a disturbing factor in everyday life. The participants described that FOF generated a feeling of vulnerability and made various activities as well as environments seem potentially hazardous. Moreover, they missed performing previous activities. FOF was a varying experience, incited and sustained by an awareness of falls, PD-related symptoms and disabilities, and by others in their environment. The persons with PD adopted different strategies to handle their FOF. Activities were either adapted, avoided, performed with help or carried out despite their FOF. The participants expressed their experiences of FOF solely in negative terms. This is not surprising as the words “fear of falling” implies negativity.

The participants’ experiences of FOF were associated with feelings of vulnerability and increased when being alone. However, previous quantitative studies found no significant association between FOF and living alone [2, 11]. The present study corroborates that FOF restricts participation in meaningful activities [9]. Although one previous study of persons with PD did not report FOF as impacting on their decision to engage in physical exercise [30], another study showed that FOF is perceived as a barrier to exercise [31]. The latter might result in that individual, non-supervised exercises are not performed although prescribed. Activities in groups or together with others might increase the possibilities for persons with PD to participate in exercise. In fact, a previous study of persons with PD as well as the European physiotherapy guidelines for PD highlight the effectiveness of supervised training as compared to non-supervised home exercises as a method to deliver interventions [32, 33].

The participants described that their FOF made a variety of activities and environments seem hazardous and daunting. For example, floors that looked slippery, even if they were not, increased their FOF. This indicates that environmental design may be of importance in relation to FOF. Almost all participants in the present study also expressed an increased FOF while climbing stairs, which has previously been reported in three studies of persons with PD [3, 34, 35]. However, stairclimbing has been considered non-problematic for persons with PD, as the steps are hypothesized to act as external cues and thereby facilitate gait [36]. In a study of walking difficulties in everyday life, the participants with PD did in fact rate stairclimbing as one of the easiest activities [37]. Taken together, these findings suggest that even though persons with PD might be physically able to climb stairs, stair climbing can be associated with an increased FOF and may therefore benefit from special attention in clinical practice.

Many of our participants expressed a concern how to get up after a fall. This is in agreement with a previous study of persons with PD [9]. Such hands-on knowledge has been requested also by caregivers of persons with PD who repeatedly fall [38]. This seems to be a common concern for persons with FOF, since it is expressed also in qualitative studies of FOF among stroke survivors [17] and persons that have had a hip fracture [18]. In order to reduce FOF, teaching persons with PD how to get up from the floor has previously been recommended [39]. The present study emphasizes that this is of importance.

FOF seemed to be affected by one’s own as well as by other persons’ experiences of falls. A previous study targeting elderly persons without PD showed that although their FOF was closely linked to experiences of falls, it was not initially affected by other person’s falls [21]. In our study, experiences of FOF were described by men as well as women, and by those with and without a history of falls. This supports previous findings which showed that gender and experiences of falls are not independently associated with FOF in people with PD [2, 4, 10, 11, 13], which is in contrast to studies that involve elderly persons without PD [14].

Our participants expressed that their FOF increased as a result of various PD-related symptoms and disabilities, such as walking difficulties, balance problems, wearing off episodes and freezing of gait. This supports findings from previous quantitative as well as qualitative studies of persons with PD [2, 4, 10–13, 40, 41]. Similarly, post-stroke changes (e.g., dizziness, reduced balance and mobility) have been found to increase FOF among stroke survivors [17].

The participants described various strategies to handle their FOF. Some persons described that they carried out activities despite of their FOF because they valued the activity. In previous studies of elderly persons without PD, participants have expressed that one should not think about the FOF and that you have to get on with your life and conquer the fear [19, 20]. Finding ways to utilize intrinsic motivation like this might be beneficial in interventions addressing FOF. Such interventions should promote safe activity performance as risk taking behavior may increase the risk of falls.

To disregard FOF and continue activity performance, as reported in our study, might be a way to handle FOF and sustain quality of life. Since engaging in meaningful activities can be a way for persons with PD to sustain their quality of life [42], persons with PD who experience FOF may need guidance and support from health-care professionals as well as from family and friends to be able to continue their treasured activities in a safe way.

Several of our participants described that assistive devices reduced FOF although these devices were not always perceived to be entirely safe. In a previous study, caregivers of persons with PD who repeatedly fell highlighted that a walking device can be a risk factor for falls if the person with PD cannot handle it properly [38]. Another study of persons with PD revealed that many falls among repeated fallers occurred while using a walking device [43]. Taken together, persons with PD might benefit from learning how to use their walking devices, and the health care system ought to make sure that the devices are suitable and safe. Efforts are also needed to develop assistive devices that better address the needs of persons with PD.

FOF is a complex phenomenon without a single, clear definition. Although several studies have shown that FOF and falls are not independently associated in PD [2, 4, 10, 11, 13], our participants had obvious difficulties in separating FOF from actual falls, and efforts to decrease FOF from efforts to decrease the risk of falls. The interviewer had to remind the participants about the focus of the interviews, i.e., FOF. This challenge is reflected also in qualitative studies of FOF in elderly persons without PD, which sometimes describes efforts to avoid falls and effects of falls instead of FOF [19–21].

There are many similarities between the experiences of FOF as described by our participants and those described by elderly persons without PD [19–22]. One previous study of elderly persons without PD described two main strategies for handling falls and FOF: exercising precaution and striving for independence. These two strategies included sub-strategies, such as eliminating hazards, restricting activities, running the risk, and using assistive devices [22]. These strategies are similar to those described by our participants. The most prominent difference between the experiences of FOF in persons with and without PD seem to be the effect of PD-related symptoms in persons with PD. Our participants described, primarily in the second theme, how various PD-specific disabilities affected their FOF, such as wearing off episodes and freezing of gait. Indeed, persons with PD have specific problems related to, e.g., balance and walking, which seem to cause specific challenges when dealing with FOF.

### Strengths and limitations

The selection of participants was performed in several steps, in order to ensure the inclusion of information rich cases and to capture a variation in experiences of FOF, which strengthens the credibility of the study [29]. That is, the participants were selected based on survey data, which was validated through phone interviews. Recruitment was performed gradually as an effort to achieve heterogeneity in the study sample. The spread in age, sex, PD duration, PD-related symptoms and disabilities implies that our sample is acceptably heterogeneous. The study sample included persons without experiences of previous falls during the past six months as well as single-fallers and frequent fallers. The study does not include any descriptive data on the presence or absence of freezing of gait among the participants; future studies targeting FOF should preferably include this, as these phenomena are associated [40].

The interviewer finalized each interview by summarizing the main points and gave the participants the opportunity to make clarifications, but the interpreted meaning (categories and themes) was not checked with the participants. However, all four authors took an active part in finalizing categories and themes, in order to ensure that they emerged from the data. Thus, there was a validity check within the research team, which strengthens the trustworthiness of the study [29].

## Conclusions

FOF affected the participants’ lives in several ways. It was experienced as a disturbing factor in everyday life. FOF was a varying experience and different strategies were adopted to handle FOF. The experiences of FOF were complex, multifaceted and varied over time and in relation to different activities and environments. This indicates that interventions targeting FOF need to be individually tailored for persons with PD and focus on several aspects, e.g., PD-related symptoms and disabilities, activities and environmental factors. Interventions might benefit from including walking-related activities in various environments (with and without the use of walking devices), stair-climbing and teaching persons with PD how to get up from the floor, but also finding strategies to handle their FOF. An increased understanding of FOF is desirable among those working with people with PD, as well as in society at large. This study provides new information that increases the understanding of FOF, which has implications for researchers as well as clinicians working with persons with PD and FOF.

## Additional file

Additional file 1: The study-specific semi-structured interview guide. (DOCX 13 kb)

## Abbreviations

FOF: Fear of falling
PD: Parkinson’s disease
