# Supplementary material for: Experiences of fear of falling in persons with Parkinson’s disease – a qualitative study
Source: BMC Geriatr. 2018 Feb 6;18:44. doi: 10.1186/s12877-018-0735-1 (PMC5801775; doi:10.1186/s12877-018-0735-1)
Supplement: Additional file 1: — The study-specific semi-structured interview guide. (DOCX 13 kb) [file 12877_2018_735_MOESM1_ESM.docx]

**Additional file**

The study-specific semi-structured interview guide:

- What does it mean to you to be afraid of falling?
- When are you afraid of falling? (Activities, environments, influences from internal/ external factors)
- How has your fear of falling developed over time?
- Why do you think that you are afraid of falling; what are you afraid of?
- What would make you less afraid of falling in these situations?
- Is the intensity of your fear of falling constant or does it vary (with time of day, indoors/outdoors, how you feel at the moment, whether you are alone/have company, etc.)?
- Have you made any changes or adjustments in your life due to your fear of falling?
- Do you behave differently due to your fear of falling?
- Would your life be any different if you were not afraid of falling?

Follow-up questions and probes were used to deepen the participants’ answers.
